# Hyperimmune yolk extract with Immunoglobulin Y basic active principle as a possible adjuvant treatment in patients who need/benefit from neurorehabilitation, with *Clostridium difficile* (*Clostridioides difficile*) enterocolitis as intercurrent comorbidity – a systematic literature review

**DOI:** 10.25122/jml-2021-0301

**Published:** 2022-02

**Authors:** Mihaela Mandu, Gelu Onose

**Affiliations:** 1. Department of Physical and Rehabilitation Medicine, Faculty of Medicine, Carol Davila University of Medicine and Pharmacy, Bucharest, Romania; 2. Neuromuscular Rehabilitation Clinic Division, Emergency Teaching Hospital Bagdasar-Arseni, Bucharest, Romania

**Keywords:** *Clostridium difficile*, immunoglobulin Y, yolk extract, IgY, enterocolitis

## Abstract

The study aims to add a new and beneficial perspective using Immunoinstant G food supplement as an adjuvant treatment. It is essential to study the bibliographic resources in the field to identify the current stage of knowledge on this topic. For this purpose, we have prepared a systematic literature review, focusing on the possibilities of improving the treatment of *Clostridium difficile* (*Clostridioides difficile*) enterocolitis in patients who need/benefit from neurorehabilitation. The systematic literature review was prepared using the Preferred Reporting Items for Systematic Reviews and Meta-Analyses (PRISMA). We obtained a number of 6 articles that were considered in the elaboration of our systematic literature review. We identified that this field is insufficiently studied and needs additional clinical trials. Our study contributes to increasing this understanding based on the thorough theoretical and practical approach of this topic.

## Introduction

*Clostridium difficile* is a Gram-positive, anaerobic, spore-forming bacterium. It was first isolated in 1935, by Hall & O’Toole, as Bacillus difficile. Due to the morpho-functional and biological differences between this bacterium and others from the genus Clostridium, in 2016, the bacterium was transferred from the Genus Clostridium to the Genus Clostridioides and received a new name: *Clostridioides* *difficile* [1]. However, given the existing similarities between the latter and the bacteria of the genus Clostridium, the common abbreviation C.diff was preserved [1]. Recent studies reveal that the microbiota plays an important role in immunity [2]. The intestinal microbiota is influenced by diet and compounds in food products [3]. Before the bacterium can affect a host, the pathogen must colonize it [4]. The spores enter the body through the fecal-oral route, in poor hygiene conditions, directly, from patient to patient, or through contact with various materials, including potentially spread by the medical staff [5]. The contamination occurs under prolonged hospitalization conditions, when the germ spores remain viable on various objects from the hospital, as they are very resistant and persist in the environment for long periods [5].

Over 80% of the deaths caused by *Clostridioides difficile* infection occur among people over 65, with old age being one of the main risk factors for *Clostridioides difficile* infection (CDI), along with the use of pro clostridial antibiotics [6, 7]. The bacterium has a surface layer composed of proteins, the so-called “Surface Layer Proteins (SLPs)”, which plays an important part in modulating the host’s immune response [8]. *Clostridioides difficile* produces two toxins: Enterotoxin (toxin A) and Cytotoxin (toxin B). Toxins A and B (TcdA and TcdB) are two high molecular weight exotoxins consisting of a polypeptide chain. It is considered that glucosyltransferase present in Toxin B is responsible for the changes in the cell and symptom occurrence in case of infection with *Clostridioides difficile* [9]. The devastating effect of the bacterium on the intestinal mucosa appears because, once the toxins reach the cytoplasm, they affect the cytoskeleton, thus leading to the degradation of the tight junctions among the intestinal cells with the loss of epithelial integrity and the occurrence of “pseudo-membranes” [10].

Because the bacterium *Clostridioides difficile* usually colonizes the gastrointestinal tract, the infection only occurs when the bacterium produces toxins. Therefore, the etiologic diagnosis is based on the detection of these toxins. The non-toxigenic strains are not considered pathogenic and thus, are not perceived as causes of enterocolitis. The toxins can be isolated directly from feces or cultures made from feces [11]. The most widely used techniques for laboratory diagnosis are the detection of Glutamate Dehydrogenase (GDH), the detection of toxins A/B, respectively of the tests for the quantification of these toxins [12], the detection of the binary toxin (cytotoxic and immune-enzymatic tests), PCR. The current standard treatment of Clostridioides enterocolitis is hydro-electrolytic rebalancing, anti-diarrheic diet, cessation of antibiotic therapy, starting treatment with Metronidazole, in the mild stages of CDI, and Vancomycin in the moderate and severe stages [13, 14]. If so far, Vancomycin has only been administered in severe cases of the disease and was considered “a major error of treatment in case of mild stages of CDI” [11], a recent study published in 2019 highlights that orally or enterally administered Vancomycin is preferable in the first-line antimicrobial treatment for all disease stages [14]. Another therapeutic option is fecal transplantation; its efficacy in the case of diarrhea induced by antibiotic therapy was proven even before C. Diff. bacterium was identified as an etiological agent [10]. The derivatives of fecal microbiota can be administered orally [10]. There are antibodies (AB) against the surface structures of the bacterium, as well as against the toxins produced by *Clostridioides difficile* [4]. There are several types of AB against the surface structures of *Clostridioides difficile*. Most of them are orally administered immunoglobulins G (IgG), harvested from cattle and rabbits [16, 17]. The AB produced by *Clostridioides difficile* against the toxins acts by blocking the RBD domain of the bacterial toxin, thus leading to the inhibition of the toxin binding the receptors on the surface of intestinal cells. Therefore, the endocytosis of the toxin is inhibited [4]. The most important types of AB against the toxins used so far in anti clostridial immunotherapy are immunoglobulins Y(IgY) and immunoglobulins G (IgG) [4]. “In the last decades, the basic technology of avian antibodies has gained more ground in the medical field, successfully replacing the immunoglobulins obtained from mammals. Immunoglobulin Y (IgY) is extracted from hyperimmune egg yolk harvested from hens (Gallus domesticus) immunized with various antigens [...] and the formulation of some therapeutic products for the administration on human subjects with clinically symptomatic infections determined by Rotavirus, Herpes Simplex type 1 virus, Human Papillomavirus or herpes zoster virus” [18].

In the last two decades, bacterial resistance to antibiotics has continually increased, becoming one of the most important public health problems worldwide [19, 20]. The use of antibiotics in animal rearing is also responsible for this problem [21]. Moreover, due to the use of antibiotics, the rate of the post-infection survivors increased, most of them remaining with morpho-functional deficits, thus requiring consecutive rehabilitation treatment [22]. The pathology that requires/benefits from neurorehabilitation and the neuro-bio-pathologic state (including comorbidities and/or complications) debilitates the patients even more, making them more susceptible to the infection with *Clostridioides difficile*. Neurologic patients are often severe cases, who, due to the (poly-)traumatic context and/or degenerative neurovascular pathology and/or some neoplastic etiology and/or some neuro-infections etc, have few biological resources, including from the point of view of immune defense [23]. Thus, these are added to the underlying neurologic/neurosurgical suffering, which in the context of the well-known morpho-(dys-)functional immune-neuro-endocrine inter-relations predispose even to disturbances of the immune function [23].

Moreover, due to the frequent infectious complications that these patients inherently have, the administration of antibiotics is often necessary (due to neuro-somatic and consecutive visceral dysfunctions; respiratory disorders, deglutition disorders, urinary control disorders). Therefore, most of the time, these patients require mechanical ventilation, in acute cases, and subsequently tracheostomy, with tracheal cannula insertion, respectively gastrostomy and/or fixed urinary probe – all these being entrance gates for the infectious agents). Furthermore, another important factor that increases the risk of *Clostridioides difficile* enterocolitis in such patients is intestinal stasis due to intestinal paresis and immobilization syndrome. Under these conditions, it is necessary to identify adjuvant treatments for conventional antibiotic therapy in these patients. Therefore, we aim to conduct a study on the efficacy of treatment with Immunoglobulin Y (IgY) extracted from hyperimmune egg yolk in patients needing neurorehabilitation, infected with *Clostridium difficile*, to reduce the recurrence rate and severe forms of the disease. Before initiating this study, we will conduct a systematic literature review.

## Material and Methods

**Figure 1.**
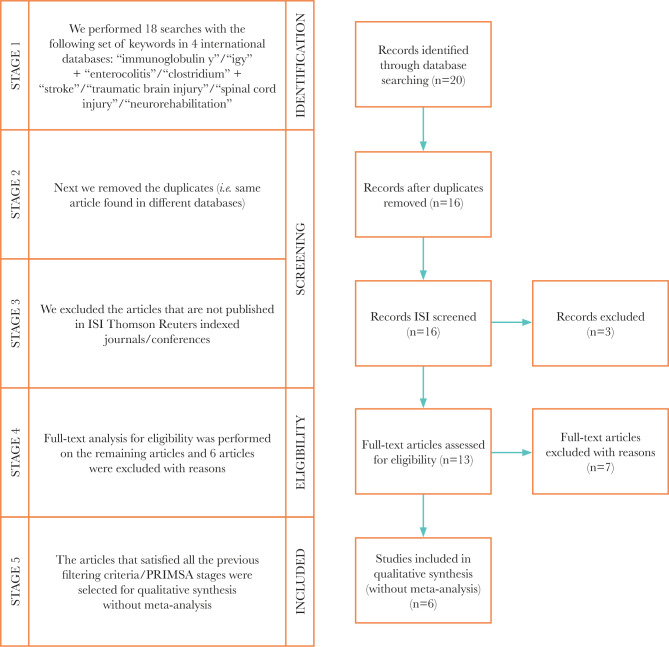
The PRISMA diagram for the study selection process.

It is essential to study the bibliographic resources in the field to identify the current stage of knowledge on this topic. For this purpose, we have prepared a systematic literature review according to the famous method of filtration/selection of the material in the field, widely used and accepted worldwide: Preferred Reporting Items for Systematic Reviews and Meta-Analyses (PRISMA) (Figure 1) More precisely, we made comprehensive searches of COCHRANE, NCBI PubMed, NCBI PMC, ELSEVIER, ISI Web of Knowledge/Sciences, PEDro (Physiotherapy Evidence Database) to check whether the articles selected according to the keywords (see below) are indexed in this international medical databases. The first consultation took place in December 2019 according to the following keywords/combinations (/“syntaxes”): [“*clostridium difficile”*, “enterocolitis”, “immunoglobulin y”], [“clostridium”, “enterocolitis”, “immunoglobulin y”], [“*clostridium difficile”*, “enterocolitis”, “IgY”], [“IgY”, “enterocolitis”, “clostridium”]. Two articles resulted, out of which only one matched our topic (Appendix 1). We have very recently resumed the search, using the same databases, with an extended panel of keyword/combinations (/“syntaxes”) (Table 1) on a period of 11 years (Jan 1, 2010 – Dec 31, 2020). After taking all the filtration/selection steps through the PRISMA method, we retrieved a number of 6 articles that matched our topic (Table 2). We included open access articles written in English and published in ISI-indexed journals in the filtration/selection process. We also included in the database several works freely identified in other bibliographical resources, including the Romanian profile literature, in addition to the ones resulting from the PRISMA selection method.

**Table 1.** Keywords/combinations (/“syntaxes”) of keywords used for international medical databases search.

	**Elsevier**	**PubMed**	**PMC**	**PEDro**	**Total**
“IgY” + “enterocolitis” + “stroke”	0	0	3	0	3
“immunoglobulin y” + “enterocolitis” + “stroke”	0	0	0	0	0
“IgY” + “enterocolitis” + “traumatic brain injury”	0	0	0	0	0
“immunoglobulin y” + “enterocolitis” + “traumatic brain injury”	0	0	0	0	0
“IgY” + “enterocolitis” + “spinal cord injury”	0	0	0	0	0
“immunoglobulin y” + “enterocolitis” + “spinal cord injury”	0	0	0	0	0
“IgY” + “enterocolitis” + “neurorehabilitation”	0	0	0	0	0
“immunoglobulin y” + “enterocolitis” + “neurorehabilitation”	0	0	0	0	0
“IgY” + “clostridium” + “stroke”	0	0	5	0	5
“immunoglobulin y” + “clostridium” + “stroke”	0	0	0	0	0
“IgY” + “clostridium” + “traumatic brain injury”	0	0	0	0	0
“immunoglobulin y” + “clostridium” + “traumatic brain injury”	0	0	0	0	0
“IgY” + “clostridium” + “spinal cord injury”	0	0	0	0	0
“immunoglobulin y” + “clostridium” + “spinal cord injury”	0	0	0	0	0
“IgY” + “clostridium” + “neurorehabilitation”	0	0	0	0	0
“immunoglobulin y” + “clostridium” + “neurorehabilitation”	0	0	0	0	0
“IgY” + “enterocolitis” + “clostridium”	0	0	9	0	9
“immunoglobulin y” + “enterocolitis” + “clostridium”	0	0	3	0	3
Total	0	0	20	0	20

**Table 2.** Articles selected through to the PRISMA method.

**No.**	**Article**	**Link**
1	Poster Sessions, Immunology. 2012 Sep; 137(Suppl 1): 185–772. Published online 2012 Sep 12. doi: 10.1111/imm.12002	link
2	Xiang-He Lei, Barry R. Bochner – Using Phenotype MicroArrays to Determine Culture Conditions That Induce or Repress Toxin Production by Clostridium difficile and Other Microorganisms, PLoS One. 2013; 8(2): e56545. Published online 2013 Feb 20. doi: 10.1371/journal.pone.0056545	link
3	EMA Committee for Medicinal Products for Veterinary Use (CVMP) and EFSA Panel on Biological Hazards (BIOHAZ), David Murphy et al. – EMA and EFSA Joint Scientific Opinion on measures to reduce the need to use antimicrobial agents in animal husbandry in the European Union, and the resulting impacts on food safety (RONAFA) – EFSA J. 2017 Jan; 15(1): e04666. Published online 2017 Jan 24. doi: 10.2903/j.efsa.2017.4666	link
4	Greg Hussack, Jamshid Tanha – Toxin-Specific Antibodies for the Treatment of Clostridium difficile: Current Status and Future Perspectives – Toxins (Basel) 2010 May; 2(5): 998–1018. Published online 2010 May 7. doi: 10.3390/toxins2050998	link
5	Mia Strom, Tamsyn Crowley, Sarah Shigdar – Novel Detection of Nasty Bugs, Prevention Is Better than Cure – Int J Mol Sci. 2021 Jan; 22(1): 149. Published online 2020 Dec 25. doi: 10.3390/ijms22010149	link
6	Terry W. Bilverstone, Megan Garland, Rory J. Cave et al. – The glucosyltransferase activity of C. Difficile Toxin B is required for disease pathogenesis – PLoS Pathog. 2020 Sep; 16(9): e1008852. Published online 2020 Sep 22. doi: 10.1371/journal.ppat.1008852	link

## Results

After going through all the filtration/selection steps using the PRISMA method (Figure 1), we selected 6 contributive articles for our topic between 01.01.2010–31.12.2020 (Table 2). Of the 6 articles selected, only one is directly related to the topic we investigated. None refers to the study of the effectiveness of IgY treatment in patients with *Clostridium difficile* hospitalized in the neuromuscular rehabilitation wards. Therefore, we identified that this niche is insufficiently studied and needs additional clinical trials in this field. Thus, our study contributes to increasing this understanding based on the thorough theoretical and practical approach of this topic.

## Discussion

*Clostridium difficile* infection poses major problems within our clinic division, a general aspect in most clinic divisions of neuromuscular rehabilitation. Moreover, nosocomial infections are a real problem in hospitals worldwide [24]. Hence, the need to find alternative treatments to optimally manage the epidemiologic situation in hospitals is objectified, taking into account the hospital infrastructure in Romania. Treatment with immunoglobulin Y is among the insufficiently developed therapeutic options. There are data in the specialized literature that objectify its superiority compared to antibiotic therapy to eliminate the recurrent episodes of disease associated with the infection with *C. difficile* [4]. Immunotherapy is a growing field that requires many studies.

We hope that this will contribute to the decrease of severe cases or relapses with this comorbidity, especially in the several neurologic patients admitted to our Neuromuscular Rehabilitation Clinic Division.

## Conclusion

Due to the development in the last decade of some strains of *Clostridioides difficile* with increased virulence compared to the previous ones and resistance to classical antibiotic therapy, the need to optimize the treatment plans is required, including through the identification of possible alternative/complementary treatments.

Thus, the studies regarding immunotherapy in *Clostridium difficile* infection became more and more commonly used. However, following this systematic review using PRISMA, we concluded that there are few studies in the literature on the efficacy of immunoglobulin Y treatment in patients with *Clostridium difficile* and no studies on its use as an adjuvant treatment of hyperimmune yolk extract with Immunoglobulin Y as a basic active principle, in patients who need/benefit from neurorehabilitation and have enterocolitis with *Clostridium* *difficile* (*Clostridioides difficile*) as intercurrent comorbidity.

## Acknowledgments

### Conflict of interest

The authors declare no conflict of interest.

### Personal thanks

We would like to thank Assoc. Prof. Eng. Vlad Ciobanu, Ph.D., for his valuable contribution and support for providing the computer infrastructure to conduct our systematic review of the literature.

### Authorship

GO contributed to conceptualizing and the methodology of this literature review. MM contributed to writing the original draft and editing the manuscript.

## Appendix

### Appendix 1. Table with the article selected after the first interrogation of the medical databases (December 2019).

**Table:**

No.	Article	Link
1	Greg Hussack, Jamshid Tanha – Toxin-Specific Antibodies for the Treatment of Clostridium difficile: Current Status and Future Perspectives – Toxins (Basel) 2010 May; 2(5): 998–1018. Published online 2010 May 7. doi: 10.3390/toxins2050998	link
